# Recognition of Barriers to Physical Activity Promotion in Immigrant Children in Spain: A Qualitative Case Study

**DOI:** 10.3390/ijerph16030431

**Published:** 2019-02-02

**Authors:** Romain Marconnot, Antonio Luís Marín-Rojas, Jose Manuel Delfa-de-la-Morena, Jorge Pérez-Corrales, Javier Gueita-Rodríguez, Cesar Fernández-de-las-Peñas, Domingo Palacios-Ceña

**Affiliations:** Department of Physical Therapy, Occupational Therapy, Physical Medicine and Rehabilitation of Universidad Rey Juan Carlos, Avenida Atenas s/n, 28922 Alcorcón, Spain; romain.marconnot@urjc.es (R.M.); antonio.marin@urjc.es (A.L.M.-R.); jose.delfa@urjc.es (J.M.D.-d.-l.-M.); jorge.perez@urjc.es (J.P.-C.); javier.gueita@urjc.es (J.G.-R.); cesar.fernandez@urjc.es (C.F.-d.-l.-P.)

**Keywords:** emigrants and immigrants, exercise, child, parents, qualitative research

## Abstract

Physical activity facilitates the acquisition of healthy habits from childhood to adulthood. Differences exist regarding the performance of physical activity among immigrant children compared to native Spanish children. The purpose of the study was to describe the barriers that exist for the promotion of physical activity. A qualitative case-study approach was implemented. Parents of immigrant children, teachers, a school principal, and priests were included, using purposeful sampling. Data were collected from 25 participants, via unstructured and semi-structured interviews, focus groups, and researchers’ field notes. A thematic analysis was performed and ecological levels were identified. Our findings revealed the following barriers to performing physical activity: (a) the meaning of physical activity, (b) gender inequalities, (c) academic burden, (d) lack of social contact, (e) expenses and family economy, (f) lack of infrastructure and natural surroundings, (g) time constraints, (h) fear and insecurity, and (i) the reason for immigrating. These results may be used to revise the school curriculum, promoting equal opportunities for physical activity and encouraging family participation. Additionally, urban design policies should be encouraged to facilitate access to open spaces for recreation within cities.

## 1. Introduction

Physical inactivity is considered the fourth leading risk factor for global mortality [1]. On a global level [2], more than 80% of school age youth are physically inactive. More specifically, in Spain, gender differences have been documented, with girls being overall less active than boys (16.3% vs. 8.2%) [3,4]. To counteract this problem, regular physical activity is recommended as it provides numerous health benefits, such as reductions in hypertension, coronary heart disease, type 2 diabetes, breast cancer, and stroke [5]. Additionally, physical activity improves body weight control and promotes muscle and joint functioning [5], improves concentration, and enhances children’s school performance [6,7].

A number of factors have been shown to influence physical activity levels in children and adolescents. These include the school environment, immigration, culture, and the social and community context where the child lives [8,9,10,11,12,13]. The school environment plays an important role in the promotion of physical activity, thanks to physical education and the organization of after school activities that promote the practice of sports, games, and other physical activities [7,11,14]. In Spain, the 2012 national health survey [4] documents differences between people who were born in Spain and those who were born abroad but subsequently immigrated to Spain. For people over the age of 15, 43.8% of those born in Spain (i.e. the native population) were sedentary, compared to 48.3% of people born abroad (i.e. the immigrant population) [4]. This finding is supported by previous studies [9,10] that show that children of foreign parents are less physically active.

Ethnicity and culture are important determinants with regard to the performance of physical activity in immigrant children. In a study of multi-ethnic parents’ views of children’s physical activity in North West England, Trigwell et al. [10] reported that on an environmental level, barriers to physical activity included safety concerns, and both a lack of resources and lack of access. Additional barriers were noted for ethnic groups with a Muslim faith (Asian Bangladeshi, black Somali, Yemeni), who reported a lack of culturally appropriate physical activity opportunities for girls. In addition, the perspective of immigrant parents regarding physical activity may condition the performance of the same in their children. For example, parents can positively influence their offspring and act as role models, by providing emotional support, economic resources (covering expenses), and by providing transportation with their car [7,12]. In contrast, parents may also limit their child’s physical activity when they perceive that insufficient safety conditions are available for the performance of physical activity [7,10,13], or when they feel that the curricular content of the child should be oriented towards mental-cognitive training as opposed to physical education [15]. 

Where an immigrant child lives also impacts their level of physical activity. Aspects such as the presence of a means of transport, sports centers, the level of safety and crime in the neighborhood and the presence of parks are all relevant when performing physical activity, via games and sports, and/or allowing children to walk to school on their own [7,12,15]. Lin et al [16], in their study about neighborhood social environments and children’s independent mobility, described how children with parents who perceived their neighborhood as being safe and more cohesive, and who lived closer to their child’s school, were more likely to allow them to travel to school unaccompanied. For New Zealanders, European, Māori, Samoan, and other Pacific parents in Australia [16], and also for Asian minority parents in London, and Turkish minority parents in Germany [17], security, danger, and crime were common concerns that impacted allowing their children to go out alone.

However, these aforementioned factors do not act independently of each other. Indeed, certain environments (social and physical environments) and policies, can make a community and its environment either more favorable or more adverse toward the promotion of physical activity [18]. To better understand this relationship between individual and environmental factors, ecological models [18] focus on the relationship between people and their physical and sociocultural environments and show that environmental contexts are predictors of health behaviors, such as physical activity [18]. Ecological models incorporate a wide range of influences at multiple levels. Levels of variables or dimensional studies included in ecological models of physical activity include intrapersonal (biological, psychological), interpersonal/cultural, organizational variables, or those related to the physical environment (built, natural), and policy (laws, rules, regulations, codes) [18]. Ecological models are particularly useful in assisting research and practice when applied to a specific behavior since unique environmental and policy factors may influence each behavior [2]. Ecological models identify factors and design interventions to influence multiple levels, including individuals, social environments, organizations, built environments, and policies, in order to achieve population improvements in public health [18]. 

Despite accumulating evidence of factors that promote physical activity among immigrant children, there is little research on the parents’ perspective and that of other physical activity stakeholders (teachers, community leaders). In Spain, a gap exists in the identification of barriers and/or difficulties for the promotion of physical activity, both from the parents’ perspective and that of the members of the social and community environment, such as teachers, school principals, and social community leaders.

The purpose of our study was to describe barriers for the promotion of physical activity, perceived by parents, teachers, a school principal, and social community leaders. The question that guided our study was: What barriers do parents and teachers encounter when promoting physical activity among recently immigrated children who face a new cultural context?

## 2. Materials and Methods

The guidelines for conducting qualitative studies established by the consolidated criteria for reporting qualitative research [19] and the Standards for Reporting Qualitative Research (SRQR) [20] were followed. Qualitative methods are useful for understanding the beliefs, values, and motivations that underlie individual health behaviors [21]. Furthermore, qualitative studies have been used to study parents’ perceptions of physical activity and to identify strategies to incorporate into the prevention of childhood diseases [22]; to explore the links between parents´ and children’s perspectives on culture and healthy lifestyle behaviors, such as physical activity [10,22]; and to examine social neighborhood environments and children’s mobility [16], and the process for increasing physical activity among elementary-aged children [23]. The design and manuscript structure were inspired by qualitative studies reported by Palacios-Ceña et al. [24].

### 2.1. Study Design

A qualitative descriptive case study with embedded units was conducted [21,25]. A case study may be formed of different units, which help to describe a phenomenon. These units may be different participants from different contexts and places who are only connected by the phenomenon under study [21,25,26]. In this study, the phenomenon under study was the barriers for the promotion of physical activity in immigrant children, perceived by different participants, including parents, teachers, a school principal, and social community leaders.

### 2.2. Context 

This study was conducted in the city of Alcorcón (Madrid, Spain), with a population of 175,000 inhabitants in the year 2017. The reported immigrant population rate of Alcorcón was 11.9% in 2017, compared to an average 13% in the community of Madrid [27]. The reported population of children between four and 15 years of age in 2014 was 17,765, of which 1,883 were foreigners, representing 10.5% of the child population. We reached a consensus to include participants from two contexts: (a) the school environment, by including two educational centers (EC) from Alcorcón; and (b) the social-community environment via the inclusion of two Catholic parishes (CP) of Alcorcón. 

### 2.3. Participants

We included all those who met the inclusion criteria and who participated in the promotion of physical activity among immigrant children at the school and in the social context.

#### Inclusion Criteria

- Parents: (a) living in Alcorcón (Spain) during the study, (b) who were not born in European Union countries, (c) who had children between seven and 16 years of age in the Spanish education system, and (d) whose children had been born in their country of origin.
- Teachers: (a) who at the time of study were actively working at an educational center, and (b) with over four years’ experience in teaching.
- The principal of a public, private, or charter school: (a) who, at the time of the study, was actively working at an educational center, and (b) with more than four years’ experience in teaching.
- Leaders of a community: people with a relevant responsibility post in an organization, or institution with social impact in the community where the parents lived. In this case, these were religious leaders (i.e., Catholic priests).

### 2.4. Sampling Strategies

A purposeful sampling strategy was employed [21], which involved deliberately selecting participants. In this case: people who participated in physical activity promotion for immigrant children in the school context, and/or the social context [21,25]. We contacted two EC and 10 teachers who taught children between 10–14 years of age, along with the school principal. They put us in touch with parents who met the inclusion criteria, of whom five agreed to participate in the study. Additionally, contact was made with the principals of two CP, who put the researchers in touch with immigrant parents who met the inclusion criteria, of whom seven agreed to participate in the study. Also, two priests who directed activities for the promotion of sports and cultural activities with the immigrant families agreed to participate in the study. Cumulatively, 25 participants were included in the sample and none withdrew from the study (Table 1). 

### 2.5. Data Collection

Data were collected from November 2017 until May 2018. The objective of this case study was to obtain an in-depth, multi-perspective holistic enquiry entailing the need for multiple data sources and multiple data collection tools (Table 2) [21,26]. The interviews and focus groups were conducted in Spanish and subsequently translated.

#### 2.5.1. Unstructured Interviews

Unstructured interviews were conducted first with participants 1–7. The interview started with an open question: “What is your experience with physical activity with children?” Thereafter, we listened carefully, noted the key words and topics identified in the participants’ responses and used their answers to ask for and clarify the content [21]. In this way, relevant information was collected from the perspective of the participants. A first analysis was performed on the unstructured interviews of participants 1–7. This analysis revealed relevant topics that required further study, thus making it necessary to include a second stage of data collection. 

#### 2.5.2. Focus Groups

The second stage (participants 8–25) consisted of two focus groups (see Table 2) which were based on a question guide designed to gather information regarding specific topics of interest (Table 3) [21]. 

Focus groups (FGs) were conducted to examine different perspectives within the same group, to acquire an understanding of the problems faced by the group, and to facilitate the identification of values and norms [21,28]. In total, two FGs (See Table 2) were conducted, and each FG comprised between 6–12 participants [21].

The FGs were conducted by a moderator following a uniform structure [21,28]. The moderator posed questions, based on the issues raised in the discussion, in order to further explore or clarify aspects, either on an individual level or with the group as a whole [28]. Focus groups were conducted in Spanish, and were audio recorded. Permission for these recordings was sought before the recordings began. A second analysis was performed on the two focus groups. This analysis uncovered relevant topics within the same group that required further study, thus making it necessary to include a third stage of data collection. 

#### 2.5.3. Semi-Structured Interviews

This third stage included 11 participants (parents and teachers obtained from phase 2 and the school principal and community leader obtained from phase 1) and consisted of semi-structured interviews (see Table 2) designed to gather information regarding specific topics of interest from FGs Table 4) [21]. 

#### 2.5.4. Written Documents

Field notes were taken for each of the unstructured, semi-structured interviews, and FGs (see Table 2). The researcher field notes provided a rich source of information as participants described their personal experiences, their behavior during data collection, and enabled them to note their reflections concerning methodological aspects of the data collection [21] (see Table 2).

### 2.6. Data Analysis

A thematic, inductive analysis was performed [21,26,29]. Literal transcriptions were drafted for each of the FGs, unstructured and semi-structured interviews, and researcher field notes [21]. The thematic analysis [29] consisted of identifying the most descriptive content in order to obtain meaningful units, and subsequently reduce and identify the most common meaningful groups. In this manner, groups of meaningful units were formed (i.e., similar points or content that allowed the emergence of the topics that described the study participants’ experience) [21,29]. This thematic analysis process was performed separately upon the unstructured interviews, FGs, and semi-structured interviews. Subsequently, joint meetings were held to combine the results of the analysis. Also, the data collection and analysis procedures were discussed during these meetings. In the case of differences in opinion, theme identification was performed based on consensus among the research team members. Subsequently, the research team held joint meetings to show, combine, integrate, and identify the final themes [21]. 

Once the final themes were identified, the multiple levels of the ecological model proposed by Sallis et al [18] were applied to contextualize the results of the different intervention levels towards the achievement and promotion of *active living.* Active living is a broader concept that incorporates exercise, recreational activities, household and occupational activities, and active transportation [18]. The multiple levels of influence include the following: intrapersonal factors, environmental policies (building infrastructure, land use, transportation regulations), location (access and characteristics of the places where physical activity may occur), information from the environment (including a great variety of forms of communication, providing and acquiring information), the socio-cultural environment (ethnicity, religion), and the natural environment (weather, topography, open space, air quality) [18]. At the center of the model, the person’s behavior towards the four active living domains is located, together with the person’s perception towards the different environments and the intrapersonal characteristics of each individual. No data analysis software was used.

### 2.7. Quality Criteria

The recommendations for the design of case study research in health care using the DESCARTE model [26] were followed. Also, the criteria for guaranteeing trustworthiness as cited by Guba and Lincoln were followed [30]. The techniques performed and the application procedures used to control trustworthiness are described in Table 5. These methods to increase rigor are compatible with case study designs [31].

### 2.8. Ethics

This study was approved by the Clinical Research Ethics Committee of the University Rey Juan Carlos (Project identification code: 3001201702417). The study was conducted in accordance with the principles articulated in the WMA Declaration of Helsinki (Ethical Principles for Medical Research Involving Human Subjects). Furthermore, we followed the Spanish Personal Data Protection Act and the Biomedical Research Act [32]. All participants provided written informed consent before they participated in the study.

## 3. Results

Twenty-five participants were included in the study with a mean age of 43.4 ± 8.7 years. See Table 1 for the sociodemographic profile of the participants. The teachers and the principal had 16.6 ± 8.4 years of teaching experience. The country of birth of the respective parents was Peru (4), Colombia (2), Ethiopia (1), Venezuela (1), Guatemala (2), and Senegal (2). The themes representing the participants’ experiences were extracted from the unstructured interviews, FGs, and semi-structured interviews. Nine themes emerged from the material analyzed: (a) the meaning of physical activity, (b) gender inequalities, (c) academic burden, (d) lack of social contact, (e) expenses and family economy, (f) lack of infrastructure and natural surroundings, (g) time constraints, (h) fear and insecurity, and (i) the reason for immigrating. We included the narratives taken directly from the data collection regarding the themes identified in this study.

Within the findings, the multilevel ecological model by Sallis et al. was used [18] to identify the most relevant levels of the model within the themes that were obtained. A summary of the levels of the ecological model is featured in Table 6, Table 7, Table 8, Table 9, Table 10, Table 11, Table 12, Table 13 and Table 14.

### 3.1. The Meaning of Physical Activity

One of the barriers found was related to the different perspectives concerning physical activity. For teachers, physical activity was considered part of a child’s education and linked to their relationship with their body, highlighting the need for children to acquire healthy habits through learning: “It is impossible to understand the education of these recently arrived children by separating the mental aspects from the physical ones. The body and the child’s relationship with their body should be a priority for providing healthy habits.” (teacher, focus group). Additionally, for parents, physical activity included movement, agility, physical space, the natural environment, and playing. Physical inactivity was associated with keeping the children locked up indoors: “The children live in cages, they are locked up, it is necessary to get them out of there” (mother, interview). Finally, the priests described how the children must perform activities together with the family, promoting family union as a key pillar of the community.

The participants perceived a decrease of physical activity in terms of sports and/or games. The absence of games and/or informal extracurricular activities was identified as a barrier for physical activity. The parents described the importance of games that children perform outdoors (i.e., in the street, as being an informal way to perform physical activity). They compared this with their countries of origin, and described how, in Spain, children spend more time at home: “Here, it seems like they aren’t children. They are in their home, playing with the computer, the mobile phones, they are disconnected from playing, from activity, from movement, from their childhood” (father, interview). Many times, parents and teachers concurred that movement via playing should be assigned more importance than intellectual educational pursuits: “They need to peel away from the chair and not only work with their brain, although this is also important” (teacher, interview). The levels of the ecological model identified are described in Table 6.

### 3.2. Gender Inequalities

Gender differences can represent a barrier to the performance of physical activity. Most participants narrated that no difference should exist between boys and girls and that all children should be free to select and practice their preferred activity. However, according to many parents, in real life, there was a difference. These differences existed in their countries of origin and were maintained in Spain: “Soccer is practiced by boys. There are more and more girls, but it is a boy thing. Over there, girls practice volleyball and rhythmic gymnastics. It is very rare for girls to practice it” (father, interview). The levels of the ecological model identified are described in Table 7.

### 3.3. Academic Burden

The extensive activities children perform at school in addition to homework were perceived as a barrier by parents and teachers. The school day, together with homework to be done after classes, was perceived to lead to children who were continuously tired, stressed, and who lacked time for other activities. All the parents described how the school gave more importance to time dedicated to the educational content than time dedicated to leisure. Because of this, the time dedicated to homework was a hindrance for the practice of physical activity during after-school hours: “They have a large theoretical burden, they must study and they don’t want to do anything else. The excessive amount of homework prevents them from playing and doing sports and having fun” (father, focus group). “The educational system doesn’t let the child be a child. If the school timetables were better organized, then they could have more time to play” (mother, interview). 

This point of view was shared by the teachers, who acknowledged that the homework burden should be decreased after school, and that the practice of other activities that allow children to “move” and “wake up their body” should be facilitated: “All day long they are confined to the classroom, and when they go home, they have to do their homework. When are they really children? When do they have time to jump, run, play among themselves, compete, or train any kind of sport? We are focusing on their brain and we forget that they are in a physical body which, if we do not teach them to care for it, when they become adults, they will have problems.” (teacher, interview). 

This contrasted with the point of view of the director, who felt that children must face a social and educational environment, which demands them to acquire knowledge and performance, and the school is responsible for preparing them for this: “I understand that children should play more, but, the truth is that in a few years they have to face tests and social demands. It is also our role as educators to prepare them as best as possible” (principal, interview). The levels of the ecological model identified are described in Table 8.

### 3.4. Lack of Social Contact

Participants described how contact with other native children facilitates their ability to relate with others via play and physical activity: “It is very important that they practice physical activity, to relate with others (…) they share things with school friends and they teach new things to their adopted country” (mother, interview). The teachers described how immigrant children have more difficulty interacting with native children (because they do not know the language, because of rejection, or not understanding the instructions, etc.), and participating in physical activity, therefore choosing more sedentary activities: “The less contact they have with other children, the more closed they become and they participate less in other activities and games. It’s a vicious circle.” (teacher, focus group). On the other hand, most participants agreed that physical activity in a group can help children establish a better connection with the class, with the school and the new country: “I would take away many of the classes and reinforce physical activity for newly arrived immigrant children. Movement is a way for them to communicate among themselves, it’s innate, it’s natural.” (teacher, interview).

In the construction of relationships, relatives who are believers go to church, where they share experiences, and where immigrant children have contact with other children and with Spanish families. Church worship is an opportunity to establish contact and acquire local customs: “Our role is to try and make them feel like they have a place in our family [the church]. Besides, we help them to understand our customs, we organize activities so that they begin to meet new families, so that they can integrate faster.” (priest, interview). “When we got to Spain we were disoriented, the language was the same, but we did not understand many things, here [in Spain], people behave differently, there are other ways of doing things. The church helped us to feel that we belonged somewhere. For my children, it helped them a lot because the first thing they remember is going to the camp that the church organized. They are still friends with the children they met there.” (father, interview). 

The levels of the ecological model identified are described in Table 9.

### 3.5. Expenses and Family Economy

One of the barriers is the economic costs families face when their children practice some kind of organized physical activity. The parents described how this is an expense that they cannot afford: “If you want [your child to practice sports] you have to pay and that is an expense that I cannot afford.” (father, interview). The parents described how, in their countries, there are many social subsidies for playing sports and performing physical activity: “There are associations that give you free sports training. On behalf of the town hall there are also sports centers, and everything is free” (mother, interview). The parents and teachers described the economic sacrifice the family must make in order for them to practice an activity in Spain: “I know many friends who must go to great efforts so that their sons practice soccer.” (mother, interview). This sacrifice takes many forms, such as investing considerable time, facing economic expenses to cover trips, sports equipment, and enrollment fees in the sports center. The levels of the ecological model identified are described in Table 10.

### 3.6. Lack of Infrastructure and Natural Surroundings

The teachers and parents related that the absence of sports and leisure infrastructure influences the practice of physical activity: “Our city seems like an abandoned town. There is no place to do sports. There’s not even a sports field, or a sports center to take them to.” (mother, focus group). Also, the parents associated the lack of physical activity with the excessive distance and/or poor accessibility of the space. Many of the parents lacked their own vehicle, having to use public transport as an alternative, or they lacked resources to reach the sports centers: “I would like to take my son to training every day, but we don’t have a car. And to think of using public transport, with the amount of buses that I would have to take to get there, makes it unfeasible. It’s better if he stays at home and studies other subjects.” (mother, focus group).

Many parents described that to share physical activity and games in nature with their children, they must cover long distances. Access to nature is also difficult, whereas in their countries it was part of their daily life: “In my country there were no parks, we had no need, nature was all around the house. Children were playing in the trees all day long. Getting to know ‘nature’ was not an extra activity necessary for their education. They lived with it.” (father, focus group). The levels of the ecological model identified are described in Table 11.

### 3.7. Time Constraints

Parents lacked enough time to share activities with their children because of a work timetable that was incompatible with the children’s leisure time. The workdays were long and prevented them from playing with their children outside of the home (park) or taking them to sports practices: “Work influences a lot, you aren’t with them enough [the children].” (mother, interview). “First things first, and that means working to be able to give my children an opportunity to continue forward in a new country. Games and sports, although it pains me to say it, will have to wait.” (father, focus group). From the point of view of the teachers, the organization of the child’s day hampered the practice of physical activity, as it coincided with the parents’ workday: “The extracurricular activities have timetables that are not compatible with the timetables of their parents, which are workdays of 10–14 hours each day (teacher, interview). My son had a scholarship for having come first in a sports meet at the town hall, but the timetable was from 18:30 to 19:30, and at that time I am still working.” (father, interview). 

The levels of the ecological model identified are described in Table 12.

### 3.8. Fear and Insecurity.

Perceived fear and insecurity were other hindrances for practicing physical activity. Some parents even used the term “terror” to describe their feeling of insecurity when taking their children to perform physical activity or play outdoors. They felt that it was unsafe to leave children alone playing on the street: “I am terrified about taking them to the park to play basketball. There is a big park, but it has a bit of everything and you see people who are so strange, it’s scary. And in the street the same thing happens, I don’t let them go out on their own.” (mother, interview). Some parents demanded the need for open spaces, such as parks; however, over time, many acknowledged that they stopped going to these places with their children due to feelings of insecurity: “I know that my children need to run, jump, play, practice sports and leave the home… but if the alternative is playing soccer in a neighborhood that I don’t know, or in the park with no control, I prefer that they stay at home.” (father, focus group).

In contrast, there were participants who felt that risks always exist, and therefore this must not condition children to limit their opportunities of doing sports, playing or interacting with other children and in other environments. They compared how, in their countries of origin, this risk was much more palpable, and that in Spain, children must not stop practicing sports because the risk is very different: “In my country [Columbia], there is a lot of crime. You can’t do anything in the open air. You know, kidnappings, armed assault and those kinds of things. In Spain, you go out running and you aren’t fearful of people robbing you, here you do not perceive any insecurity.” (mother, interview). The levels of the ecological model identified are described in Table 13.

### 3.9. The Reason for Immigrating.

The reasons that lead the families to immigrate can also be a hindrance for children to practice physical activity. For many parents, economic reasons were behind their choice to immigrate. Therefore, practicing physical activity was not seen as a priority: “What did we come here for (the immigrants): to work and make money, and we don’t have time to do anything more, the rest is secondary, my children understand.” (father, interview). On the other hand, when residence was more permanent (refugees, political asylum), the children’s education, with all its intellectual and physical dimensions, acquired greater importance. Children were prioritized and therefore required parents’ time: “When you realize that Spain is going to be your new home, everything changes. You try to give your children the same opportunities as any other child, for him to do what other children here [in Spain] do, to become one of them. His education is the most important thing.” (mother, focus group). The levels of the ecological model identified are described in Table 14.

Finally, Table 15 displays the levels of the ecological model identified in each of the themes. 

## 4. Discussion

Our results revealed a number of barriers for physical activity promotion in immigrant children from the perspective of parents, teachers, a principal, and community leaders. Our study continues a line of research on the factors that impact upon and influence the practice of physical activity and the implementation of a healthy lifestyle in immigrant children [10,17,22].

Previous studies regarding physical activity in children and teenagers [33,34] show how children and teenagers whose parents were immigrants practiced less physical activity compared to native children and teenagers. In the USA, Brewer et al [33] reported that this disparity is not attenuated by a child’s socioeconomic, family, or neighborhood characteristics. Also, patterns of physical activity have been found to vary according to the immigrant generation status (being born in a new country vs. being born in the country of origin), and the process of acculturation [33,34]. 

Our participants did not agree with the existence of gender inequality in the practice of physical activity; however, many parents acknowledged that this type of bias exists in their country of origin. The existence of inequality in the practice of physical activity among men and women can be considered a global phenomenon [35]. In Spain, the 2016 survey on sports habits by the Ministry of Education and Culture [36] shows that boys mostly practice sports such as soccer, cycling, and swimming, and that girls mainly practice gymnastics, hiking, and swimming. Previous studies [10,37] show that this gender difference exists even in recess hours during school, where the amount of physical activity performed varies (greater in boys) as well as the type of activities performed (“boys games” versus “girls games”). Culture, ethnicity, and religion may, in part, explain this gender difference in immigrant children [10,37]. 

Our participants perceived a significant academic burden regarding academically-oriented school activities compared to activities promoting physical skills. This is in line with Trigwell et al. [10], who reported that ethnic groups from different cultures (Asian Bangladeshi, Chinese, Yemeni) prioritized educational attainment over physical activity. In contrast, many studies [14,38] agree with the need to increase physical activity in school curricula [38] and incorporating break periods between lessons [14]. Public health recommendations highlight the pivotal role that schools and teachers play in providing equitable opportunities for physical activity for all children. Also, opportunities to increase physical activity throughout the school day should span, not only in-school time (e.g., quality recess and physical education, classroom activity breaks), but also time before school (e.g., active commuting initiatives) and after school (e.g., intramural and interscholastic sports programs) [11,38].

Our results highlight how the neighborhoods where our participants lived do not present the necessary conditions, nor do they have sufficient infrastructure, for the practice of physical activity. Previous studies [17,39] show how the neighborhood, the area of the city where they are located, the presence of parks, accessible urban transport, and comprehensive urban planning to support physical activity (walkability) all influence the performance of physical activity. More specifically, in the case of children and youth, previous studies [17,40] have shown an association between neighborhood-built environments and an increased level of physical activity (and, consequently, a decrease in sedentary time). This also applies to young immigrant children/youth of other cultures or ethnicities in Western countries [17].

Nevertheless, fear and insecurity are also major barriers for the practice of physical activity. Previous studies show how crime, insecurity, the perceived presence of “dangerous” people, and the lack of safe spaces influence the possibility of taking children to leisure activities in safe spaces (with or without physical activity) [10,13,16,17].

Previous studies [12,41] have examined the influence of parents on children’s physical activity, and the evidence for the context-specific influence of parental modelling on physical activity in childhood remains inconclusive. Along these lines, the SOPHYA Study Group [41] reported that parental modelling (physical activity practice) seems relevant for children’s physical activity (*p* < 0.001), and were stronger for children aged 10–12 years, although not to the same extent in all children. This conflicts with previous studies [12] which have shown that parental modeling was not associated with physical activity in children. We believe that an important barrier in the practice of physical activity in immigrant children is the socioeconomic-work situation of the parents, narrated in the themes “costs and economy,” “lack of time,” and the “motive for immigrating.” Parents choose to immigrate in order to earn a living and improve the family’s quality of life (via precarious employment or low-skilled employment with long working days), which is associated with insufficient economic resources and a lack of time for parents to invest in their children practicing physical activity, playing, and spending time together. Thus, a vicious circle is created, which can perpetuate certain activity routines in children in favor of sedentarism. In Spain, the National Immigrant Survey [42] describes how Latin American immigrants are labeled as economic immigrants (seeking economic improvements) who perform poorly qualified labor, and are mainly located in the lower levels of the socio-economic scale. Two out of every five immigrants are employed in manual labor and are poorly qualified (40%) and one in four (22%) performs manual labor for which a certain level of preparation is required. This means that low-skilled jobs with low salaries are perpetuated with long work days. Previous studies [22,40] show how parents’ lack of time, long work days, family burden, low salaries, and not having enough money to cover expenses (buying sports equipment, enrolling in sports activities) negatively impacts children’s physical activity. According to former studies conducted in Spain [40,43], children and adolescents who live in neighborhoods with a lower socio-economic status are less physically active.

In Spain, previous studies [44,45] that took place in the cities of Granada and Cuenca described how the volume of traffic, the poorly signposted intersections and pedestrian crossings en route to the school, and the insecurity were perceived as barriers for the performance of physical activity in children. Additionally, the organization of space, time, and physical activity performed in recess time at school was highlighted [44]. The lack of programming any physical activity during recess time led to situations where only certain physical activities were performed, monopolizing time and space, meaning that, at times, children did not participate in any physical activity and that gender differences between boys and girls was perpetuated. An example of this is soccer playing. Gutiérrez-Zornoza et al. [45] described the influence of the environment on the physical activity habits of school children. These authors reported that children perceived that residing in gated communities or in rural areas facilitated physical activity, as opposed to residing in neighborhoods and cities. They reported that their children’s feelings of insecurity regarding road traffic, car journeys, and an overall sense of not belonging or being accepted in the neighborhood were perceived as barriers to being active. Therefore, in Spain, the following barriers are highlighted: environmental barriers (accessing the schools and safe traffic), organizational barriers in the selection of physical activities within and outside the school, and social barriers (belonging to certain neighborhoods or areas). In previous Spanish studies [44,45], no distinction was made between immigrant and native children. On the other hand, within the context of Hispanic culture, in the USA, Ross and Francis [46] described physical activity perceptions, context, facilitators, and barriers from the perspective of Hispanic immigrant-origin children. These authors reported how Latino children had negative attitudes toward physical activity that were related to physical discomfort, low athletic competence, and safety concerns. Also, children had difficulties differentiating physical activity and play, and "fun" was identified as a primary driver of physical activity preferences. Also, Turner et al. [47] researched family lifestyle behaviors of Mexican-American and Mexican immigrant fathers and mothers and their repercussion on the performance of physical activities in children. This previous study found that parents were permissive regarding their child’s sedentary habits (which involved playing video games and watching television for lengthy periods), rather than actively encouraging them to be more physically active.

In our study, one might expect that immigrants who have a common language, and a common Hispanic culture (nine parents), would not encounter difficulties for promoting physical activity in their children. However, we have found similar difficulties in previous studies [10,16,17,22] that studied physical activity in children of different ethnicities, cultures, religions, and languages. This could be explained by the fact that the socioeconomic conditions for the immigrant parents of our study are perceived as a determinant for their children regarding performing physical activity, despite not having linguistic barriers. Additionally, during the process of integration, non-native children may acquire habits and customs of the new country that could influence their health, including the adoption of sedentary habits [48]. 

It is worth noting that ecological model proposed by Sallis [18] was a secondary analysis applied just to contextualize the results by classifying them. However, this ecological model was not the aim of this study despite being used with an exploratory purpose. On the other hand, this ecological model could be useful as a theoretical model to guide data collection and as a primary analysis of future qualitative studies in the field of physical activity.

The implications of this study are based on the development of specific policies, programs, and actions for fostering and promoting physical activity, as well as eliminating the associated barriers and difficulties present in the community, the school, and the homes of immigrant children and their families. These interventions must be open and acting simultaneously upon different levels. Thus, for example, the development of security policies and crime control in the physical environment (i.e., parks) of a community acts upon the levels of the policy environment, setting: access and characteristics, and perceived environment. More concretely, educational policies should be included, oriented towards improving physical education in schools, policies promoting physical activity in the community, and the use of public spaces with the development of programs oriented toward the integration of immigrant children via physical activity, policies that help immigrant families with limited resources to access infrastructures and sports resources, and finally, work policies that facilitate work timetables compatible with the leisure time of both the child and the family (policy environment level, social cultural environment level, setting: access and characteristics level, perceived environment level, and intrapersonal level). Besides, there is a need to develop programs in schools to promote equality among sexes for participation in physical activities and sports while supporting physical activity in the natural environment and open spaces (i.e., parks and forest). Likewise, it is necessary to include community initiatives that facilitate the use of leisure time outside the home for performing physical activity with other children and/or with the family. Lastly, detailed information should be provided concerning physical activity proposals by integrating public and private initiatives within the community (social cultural environment level, natural environment, information environment level, setting: access and characteristics level, behavior active living domains level, perceived environment level, and intrapersonal level). Besides, it is also important to encourage urban design policies that facilitate access to open spaces within and/or close to the communities, and the development of sports resources with accessible timetables that are compatible with the workload of parents. Also, a network of appropriate means of transport should be created to ensure access to sports centers (setting: access and characteristics level, social cultural environment level, behavior active living domains level, and intrapersonal level).

Future lines of research should study the existence of barriers for the practice of physical activity in immigrant children of different nationalities, focusing also on different sports and including girls from different cultures and ethnicities. Additionally, further research should aim to develop public policies to promote physical activity in immigrant children in urban settings. 

### Limitations

This study has several limitations. First, a small number of participants was included. The qualitative nature of this study meant that the aim was to describe the experiences of the participants and, therefore, these findings cannot be generalized. We also only included participants from a single religious context (i.e., Catholicism). It would be necessary to develop future studies with immigrants from other religions.

## 5. Conclusions

Our findings help further our understanding of the factors related with physical activity in immigrant children living in Spain. One of the strengths of this study is that this is the first study to describe the barriers for performing physical activity in immigrant children from the parents’ perspective and that of other participants living in their community and social environment. 

Despite the fact that most parents in our study came from countries with cultural and linguistic ties with Spain, they perceived barriers for the performance of physical activity that were similar to the barriers identified in immigrants without cultural or linguistic ties to other European countries.

These results may be used for the development of physical activity programs in specific social contexts. These programs should: (a) reorganize the school study plans (decreasing the educational burden and the amount of homework given to children to increase the time dedicated to physical activity), (b) promote the participation of the whole family in children’s physical activity, (c) provide economic support for families with fewer resources in order to cover enrollment costs and expenses related to children’s sports practice, (d) improve the organization of sports activities and multicultural games in the community on behalf of the town halls and public institutions, and (e) encourage the development of safe natural public spaces within cities.

Future studies should incorporate the perspective of Spanish children and adolescents of immigrant origin to provide a greater depth to the development of physical activity programs and to further understand detected barriers and facilitators for the performance of physical activity.

## Figures and Tables

**Table 1:** Sociodemographic data of participants.

Participants	Sociodemographic Data
Parents	Participants: 12 (4 women) Mean age: 47.4 ± 7.4
Teachers	Participants: 10 (5 women) Mean age: 37.6 ± 7.0 Years of teaching experience: 15.3 ± 7.6
School Principals	Participants: 1 (man) Age: 51 Years of teaching experience: 30
Community Leaders (Priests)	Participants: 2 (men) Mean age: 44.5 ± 13.4

**Table 2:** Data collection process.

Data Collection Phase	Data Collection Tool	Participants
1	7 unstructured interviews + 7 researcher field notes	2 parents, 2 teachers, 2 priests, 1 principal
2	2 focus group + 2 researcher field notes	10 parents, 8 teachers
3	11 semi-structured interviews + 11 researcher field notes	5 parents, 4 teachers, 1 principal, 1 community leader ^1^

^1^ Parents and teachers were obtained from phase 2 and the school principal and community leader were obtained from phase 1.

**Table 3:** Focus group question guide.

Phase	Contents	Time (Min)
Moderator welcome	Welcome, explanation of study aims, process of the session and rules.	5–10
Opening questions	Participants were asked about their experience with physical activity in children: Could you tell me your experience with physical activity in children?	10–20
Introductory and transition questions	The question was centered on aspects of physical activity promotion and management: What promotion activities did you perform? What activities were performed for the management of physical activity?	10–30
Key questions	Questions were posed once more on the basis of prior participant responses in order to go into greater depth regarding areas such as: Teacher–children relationships: Do you think the relationships between teachers and children may influence the promotion and management of physical activity? Parent–children relationships: Do you think the relationships between parents and their children may influence the promotion and management of physical activity? Development of physical activity programs in the school: What is your experience with programs in the school? How are these developed? How do parents’ and/or teachers’ interventions contribute towards promoting and maintaining physical activity?	20–40
Closing remarks	The moderator performed a brief summary of the contents covered. The participants confirmed whether the summary was correct and were given the opportunity to add to/modify their statement.	10–15

**Table 4:** Semi-structured interview question guide.

Research Area	Questions
Physical activity, culture and ethnic group	Do you believe that culture and/or ethnicity can influence the performance of physical activity in children? How? How do you perceive physical activity from the point of view of your culture?
Strategies for the promotion of physical activity in immigrant children	How can physical activity be promoted and fostered in immigrant children? Which strategies or interventions are most necessary?
Barriers for the performance of physical activity in immigrant children	What elements can hamper the performance of physical activity in immigrant children? What elements can do so in educational centers? What elements can do so in the community?

**Table 5:** Trustworthiness criteria applied.

Criteria	Techniques Performed and Application Procedures
Credibility	Investigator triangulation: each data source was analyzed. Thereafter, team meetings were performed during which the analyses were compared and themes were identified. Triangulation of data collection methods: including unstructured interviews, focus groups, semi-structured interviews, and researcher field notes. Participant validation: this consisted of asking the participants to confirm the data obtained at the stages of data collection.
Transferability	In-depth descriptions of the study performed, providing details of the characteristics of researchers, participants, contexts, sampling strategies, and the data collection and analysis procedures.
Dependability	Audit by an external researcher: an external researcher assessed the study research protocol, focusing on aspects concerning the methods applied and the study design.
Confirmability	Investigator triangulation and data collection triangulation. Researcher reflexivity was encouraged via the previous positioning, performance of reflexive reports, and by describing the rationale behind the study.

**Table 6:** Levels of the ecological model identified in: The meaning of physical activity.

Meaning Units of Theme	Ecological Model Level
Educational policies orientated towards academic contents compared to physical education. Insufficient programmed and planned activities available for the promotion of physical activity.	Policy environment
Participation in community events via physical activity.	Social cultural environment
Physical activity in the natural environment (park, forest, countryside) is an essential aspect for children.	Natural environment
Household: children tend to spend their time mostly in the home. Even when at home, they do not perform physical activity. Much time is spent watching the television and playing video games. Active recreation: physical activity is part of the games children play. Playing and physical activity are linked together. Occupational activities: within their own activities, the family can try and perform physical activities together.	Behavior active living domains (household, active recreation, active transport, occupational activities)

**Table 7:** Levels of the ecological model identified in: Gender inequalities.

Meaning Units of Theme	Ecological Model Level
The society identifies certain sports with a specific gender.	Social cultural environment
Active recreation: certain physical activities may be limited by gender.	Behavior active living domains (household, active recreation, active transport, occupational activities)
The environment presents difficulty for girls to access the same.	Perceived environment
The parents would like no gender barriers to exist; however, they still perceive these. Parents perceive gender barriers between boys and girls when performing physical activity in the schools of their countries of origin and this is maintained in Spain.	Intrapersonal

**Table 8:** Levels of the ecological model identified in: Academic burden.

Meaning Units of Theme	Ecological Model Level
Educational policies and excessive school activities.	Policy environment
Socially, the need for large amounts of homework is accepted, as it is considered to be beneficial for the child (being better prepared).	Social cultural environment
Household: excessive homework, which limits other activities. Active recreation: this is affected by not having time for other activities.	Behavior active living domains (household, active recreation, active transport, occupational activities)
Inappropriate organization of school work.	Intrapersonal

**Table 9:** Levels of the ecological model identified in: Lack of social contact.

Meaning Units of Theme	Ecological Model Level
The application of physical activity programs and events may help the integration of immigrant children in the new country.	Policy environment
Access to support groups and cultural and social groups facilitates the integration of immigrant children. For Catholic immigrants, the church facilitates the ability to integrate into the community.	Setting: access and characteristics
Acquiring information via other institutions, such as the church.	Information environment
Children must relate with other children in the community. Physical activity could be a good means to do so.	Social cultural environment
Active recreation: sharing games and sports among children.	Behavior active living domains (household, active recreation, active transport, occupational activities)
The teachers perceive an adverse environment for immigrant children due to language difficulties.	Perceived environment
The teachers perceive that immigrant children present greater difficulty relating with other and sharing games and physical activity with other children.	Intrapersonal

**Table 10:** Levels of the ecological model identified in: Expenses and family economy.

Meaning Units of Theme	Ecological Model Level
The creation of support programs for children, in families with limited resources in order to practice sports and/or activities.	Policy environment
The costs of some sports centers limit the access to the performance of physical activity. Ease of access due to a reduction of activity registration costs or free access.	Setting: access and characteristics
Active transport: the families help by taking their child to the physical activity; however, this entails an effort to cover the cost of the trips.	Behavior active living domains (household, active recreation, active transport, occupational activities)
The environment is perceived as a barrier for the difficulty for children to access the space.	Perceived environment
To enable the children to perform physical activity, “sacrifices” are made (money, time, transport) on behalf of the family.	Intrapersonal

**Table 11:** Levels of the ecological model identified in: Lack of infrastructure and natural surroundings

Meaning Units of Theme	Ecological Model Level
Absence of infrastructures for the practice of physical activity and sports. Sports centers or open spaces in nature that are poorly accessible because of the distance from the home. There is a lack of good transport connections to reach the sports centers or the natural or open space. Either a car is needed, or several public transport connections are needed.	Setting: access and characteristics
The natural environment and open spaces to perform physical activity are very far from the home.	Natural environment
Active transport: sports centers that are very far mean that a private vehicle is needed to reach these spaces, or several public transport vehicles.	Behavior active living domains (household, active recreation, active transport, occupational activities)

**Table 12:** Ecological model levels identified in: Time constraints.

Meaning Units of Theme	Ecological Model Level
The programming of extracurricular activities does not adapt to the family’s work constraints.	Policy environment
Access to sports centers or games is difficult because of the work timetable.	Setting: access and characteristics
Parents with a work timetable that is incompatible with playing or performing physical activity with the children in the community or sharing social and cultural events of the community	Social cultural environment
Active recreation: parents with great difficulty sharing recreational activities with their children, due to work timetables. Occupational activities: jobs with very long timetables.	Behavior active living domains (household, active recreation, active transport, occupational activities)
The parents wish they could share more time with their children and perform more activities.	Intrapersonal

**Table 13:** Ecological model levels identified in: Fear and insecurity.

Meaning Units of Theme	Ecological Model Level
The development of policies for the control of public spaces is necessary, for games, sports, and other community activities.	Policy environment
Insecurity, risk of danger, and uncontrolled environments hamper the practice of physical activity on behalf of children. These spaces are avoided by the parents, who limit activity in children.	Setting: access and characteristics
There is parental control regarding the performance of physical activity, games and sports according to the perceived risk or insecurity for their children within a community.	Social cultural environment
Participation in activities in open spaces, such as parks, is avoided because of insecurity.	Natural environment
Household activity: avoiding the performance of outdoor activities is compensated with activities in the home. Active recreation: this is affected due to the limitation of activities outside the home.	Behavior active living domains (household, active recreation, active transport, occupational activities)
Parents display fear and insecurity when leaving their children alone playing in the street or alone in open spaces. The perception of risk or insecurity in Spain is lower compared to their country of origin.	Intrapersonal

**Table 14:** Ecological model levels identified in: The reason for immigrating.

Meaning Units of Theme	Ecological Model Level
Work and making money is the family priority, limiting the access of children to sports activities and spaces. The choice to immigrate is determined by economic motives which are the family priority. In the case of temporary immigrants, physical activity is not a priority. However, for families that settle in Spain, physical activity is a necessary factor which their children must develop, like Spanish children.	Setting: access and characteristics
Occupational activities: the priority is the economy and making money.	Behavior active living domains (household, active recreation, active transport, occupational activities)
Parents who settle in Spain perceive that practicing physical activity is just another element within their education.	Intrapersonal

**Table 15:** Relationships among identified themes according to each level of the ecological model.

Ecological Model Level	The Meaning of Physical Activity	Gender Inequalities	Academic Burden	Lack of Social Contact	Expenses and Family Economy	Lack of Infrastructure and Natural Surroundings	Time Constraints	Fear and Insecurity	The Reason for Immigrating
Policy environment	X		X	X	X		X	X	
Social cultural environment	X	X	X	X			X	X	
Natural environment	X					X		X	
Information environment				X					
Setting: access and characteristics				X	X	X	X	X	X
Behavior active living domains ^1^	Hh, Ar, Oa	Ar	Hh, Ar	Ar	At	At	Ar, Oa	Hh, Ar	Oa
Perceived environment		X		X	X				
Intrapersonal		X	X	X	X		X		X

^1^ Relationship identified between theme and ecological model level (X); Household (Hh); Active recreation (Ar); Active transport (At); Occupational activities (Oa).
